# Histamine-3 Receptor Availability and Glutamate Levels in the Brain: A PET-^1^H-MRS Study of Patients With Schizophrenia and Healthy Controls

**DOI:** 10.1093/ijnp/pyae011

**Published:** 2024-02-19

**Authors:** Atheeshaan Arumuham, Matthew M Nour, Mattia Veronese, Katherine Beck, Ellis Chika Onwordi, David J Lythgoe, Sameer Jauhar, Eugenii A Rabiner, Oliver D Howes

**Affiliations:** Department of Psychosis Studies, Institute of Psychiatry, Psychology and Neuroscience, Kings College London, De Crespigny Park, London, UK; Institute of Clinical Sciences (ICS), Faculty of Medicine, Imperial College London, London, UK; Psychiatric Imaging Group, Medical Research Council, London Institute of Medical Sciences, Hammersmith Hospital, London, UK; Department of Psychosis Studies, Institute of Psychiatry, Psychology and Neuroscience, Kings College London, De Crespigny Park, London, UK; Department of Psychiatry, University of Oxford, Oxford, UK; Max Planck University College London Centre for Computational Psychiatry and Ageing Research, London, UK; Department of Information Engineering, University of Padua, Padua, Italy; Department of Neuroimaging, Institute of Psychiatry, Psychology and Neuroscience, King’s College London, London, UK; Department of Psychosis Studies, Institute of Psychiatry, Psychology and Neuroscience, Kings College London, De Crespigny Park, London, UK; Institute of Clinical Sciences (ICS), Faculty of Medicine, Imperial College London, London, UK; Psychiatric Imaging Group, Medical Research Council, London Institute of Medical Sciences, Hammersmith Hospital, London, UK; Department of Psychosis Studies, Institute of Psychiatry, Psychology and Neuroscience, Kings College London, De Crespigny Park, London, UK; Institute of Clinical Sciences (ICS), Faculty of Medicine, Imperial College London, London, UK; Psychiatric Imaging Group, Medical Research Council, London Institute of Medical Sciences, Hammersmith Hospital, London, UK; Centre for Psychiatry and Mental Health, Wolfson Institute of Population Health, Queen Mary University of London, London, UK; Department of Neuroimaging, Institute of Psychiatry, Psychology and Neuroscience, King’s College London, London, UK; Psychological Medicine, Institute of Psychiatry, Psychology and Neuroscience, King’s College, London, UK; Invicro, London, UK; Department of Psychosis Studies, Institute of Psychiatry, Psychology and Neuroscience, Kings College London, De Crespigny Park, London, UK; Institute of Clinical Sciences (ICS), Faculty of Medicine, Imperial College London, London, UK; Psychiatric Imaging Group, Medical Research Council, London Institute of Medical Sciences, Hammersmith Hospital, London, UK; H Lundbeck A/s, St Albans, UK

**Keywords:** Schizophrenia, histamine-3 receptor, neuroimaging, glutamate

## Abstract

**Background:**

The histamine-3 receptor (H3R) may have a role in cognitive processes through its action as a presynaptic heteroreceptor inhibiting the release of glutamate in the brain. To explore this, we examined anterior cingulate cortex (ACC) and striatum H3R availability in patients with schizophrenia and characterized their relationships with glutamate levels in corresponding brain regions.

**Methods:**

We employed a cross-sectional study, recruiting 12 patients with schizophrenia and 12 healthy volunteers. Participants underwent positron emission tomography using the H3R-specific radio ligand [^11^C]MK-8278, followed by proton magnetic resonance spectroscopy to measure glutamate levels, recorded as Glu and Glx. Based on existing literature, the ACC and striatum were selected as regions of interest.

**Results:**

We found significant inverse relationships between tracer uptake and Glu (r = −0.66, *P* = .02) and Glx (r = −0.62, *P* = .04) levels in the ACC of patients, which were absent in healthy volunteers (Glu: r = −0.19, *P* = .56, Glx: r = 0.10, *P* = .75). We also found a significant difference in striatal (F_1,20 _= 6.00, *P* = .02) and ACC (F_1,19 _= 4.75, *P* = .04) Glx levels between groups.

**Conclusions:**

These results provide evidence of a regionally specific relationship between H3Rs and glutamate levels, which builds on existing preclinical literature. Our findings add to a growing literature indicating H3Rs may be a promising treatment target in schizophrenia, particularly for cognitive impairment, which has been associated with altered glutamate signaling.

**Significance Statement**

Schizophrenia is a long-term condition that can be disabling to patients. Many patients continue to experience symptoms, particularly cognitive impairment, despite treatment with available antipsychotics. In this cross-sectional study, we investigate brain histamine-3 receptor (H3R) levels in patients with schizophrenia and healthy volunteers. Previous evidence has found H3R to be altered in psychotic disorders and involved in cognitive function. We compared receptor availability with brain glutamate levels and found a significant negative relationship in the cortex of patients that is absent in healthy volunteers. Our results provide the first evidence, to our knowledge, of the relationship between H3Rs and glutamate levels in humans. They highlight the potential of the H3R as a target for the treatment of notably resistant symptoms of schizophrenia, which have been associated with alterations in glutamate levels.

## INTRODUCTION

Schizophrenia and associated psychotic disorders are associated with chronic disabling symptoms and reduced quality of life (McCutcheon et al., 2020b; Jauhar et al., 2022). Nonclozapine antipsychotic agents are ineffective in treating the symptoms of approximately one-third of patients (Howes et al., 2017; Beck et al., 2019). Emerging evidence has implicated the involvement of the histaminergic system in the neurobiology of schizophrenia, particularly due to its association with cognitive processes (Esbenshade et al., 2008; Alvarez, 2009).

Histamine-3 receptors (H3Rs) act as heteroreceptors on glutamatergic neuron terminals to inhibit the synaptic release of glutamate (Molina-Hernández et al., 2001; Takei et al., 2017). H3R have been localized as heteroreceptors within the frontal cortex and the corticostriatal pathway, both of which play a critical role for cognitive processes, including working memory, attention, social cognition, and executive function (Brown and Reymann, 1996; Doreulee et al., 2001). The action of H3R on these glutamatergic circuits is thought to suppress excitatory signaling, with greater suppression impairing cognitive function (Simpson et al., 2010; Bolam and Ellender, 2016). Expanding on this, several H3R inverse agonists have been tested as treatments for cognitive impairments (Vohora and Bhowmik, 2012; Alhusaini et al., 2022). GSK-189254 reversed scopolamine-induced amnesia alongside improving various cognitive domains, including passive avoidance, water maze learning, object recognition, and odor discrimination, tasks associated with learning and memory consolidation (Medhurst et al., 2007; Foley et al., 2009). Similarly, BF2.649 (pitolisant) reduced methamphetamine- and dizocilpine-induced hyperactive locomotor activity alongside eliminating apomorphine-induced deficits in pre-pulse inhibition (Ligneau et al., 2007). Postmortem evidence has identified increased H3R concentrations in the dorsolateral prefrontal cortex of patients with schizophrenia compared with health controls and a positive correlation between cortical H3Rs and severity of schizophrenia symptoms (Jin et al., 2009). Taken together, the cognitive deficits seen in schizophrenia may in part be due to higher levels of H3R leading to reduced glutamate transmission in key brain regions.

However, the relationship between H3Rs and glutamate levels remains unclear in vivo. Thus, we aimed to investigate this in patients with schizophrenia and healthy controls. Due to preclinical evidence indicating H3R activation inhibits glutamatergic signaling, we hypothesized H3R availability would negatively correlate with markers of glutamate in the anterior cingulate cortex (ACC) and striatum for both patients with schizophrenia and healthy volunteers. Both the ACC and striatum were selected as the primary regions of interest (ROIs) based on preclinical findings of H3R’s role in glutamate release, along with being regions identified as having disrupted glutamate levels in schizophrenia (Molina-Hernández et al., 2001; Jin et al., 2009; Aquino-Miranda et al., 2016; Takei et al., 2017; Merritt et al., 2023).

## METHODS

### Ethics Statement

Approval was obtained by the West London & GTAC Research Ethics Committee (REC reference: 17/LO/1299) and the Administration of Radioactive Substances Advisory Committee (ARSAC license: 630/3764/36826). Volunteers demonstrated capacity and provided written informed consent to participate. We followed the Strengthening the Reporting of Observational Studies in Epidemiology (STROBE) reporting guidelines for case-control studies, and the study was conducted in accordance with the Declaration of Helsinki.

### Participants

Data were collected from August 16, 2018, until March 24, 2021. Two groups were recruited comprised of patients with schizophrenia and matched healthy volunteers. Patients were recruited from community mental health teams in London, United Kingdom. Patients required a diagnosis of DSM-IV schizophrenia according to the Structured Clinical Interview of DSM-IV-TR Axis I Disorders-Patient Edition (First et al., 2002). Patients with schizophrenia were classified as antipsychotic free if they had been free from antipsychotic treatment for at least 6 weeks for oral or 6 months for depot formulations (Jauhar et al., 2019). Antipsychotic naïve was defined as having had no antipsychotic treatment at all. For comparison, a sample of healthy controls that were matched for both sex and age (±3 years) was also recruited. Healthy controls were required to not have a current or lifetime history of Axis I disorder as determined by the Structural Clinical Interview of DSM-IV-TR Axis I Disorders-Patient Edition (First et al., 2002).

Exclusion criteria for all volunteers included the following: dependence on illicit substances or alcohol, positive urine drug test (SureScreen Diagnostics, Derby, UK) for any illicit substances that might affect H3R (e.g., stimulants) on the day of scanning, medical comorbidity (other than minor illnesses), current use (or use within last 3 months) of medications that modulate H3R (e.g., pitolisant), and contraindications to scanning (such as pregnancy). Specifically, all antipsychotics aside from clozapine were permitted during recruitment due to negligible affinity for H3R (Appl et al., 2012) (see supplementary Methods 1 in the supplementary information for full inclusion and exclusion criteria).

## MEASURES

### Clinical Variables

Current age and illness duration were recorded (see Table 1). Clinical symptom severity was determined using the Positive and Negative Syndrome Scale (Kay et al., 1987). Psychotropic medication histories were recorded, urine drug screens were performed, and equivalent chlorpromazine doses were calculated using the method reported by Leucht et al. (Leucht et al., 2014).

**Table 1.** Demographics and Experimental Variables

Demographics and experimental variables	Healthy volunteers (n = 12)	Patients (n = 12)	*P*
Sex, male: female	9:3	9:3	1.00
Age, y, mean (SD)	30.3 (11.5)	30.4 (13.0)	.80
Weight, kg, mean (SD)	73.1 (12.4)	79.7 (13.4)	.22
Injected dose, MBq, mean (SD)	275.6 (10.7)	251.5 (18.1)	<.01
Illness duration, y, median (IQR)		3.0 (7.0)	
Antipsychotic free, n (%)		7 (58.3)	
Chlorpromazine equivalent dose/mg, mean (SD)		324.3 (118.2)	
PANSS positive, mean (SD)		16.8 (3.7)	
PANSS negative, mean (SD)		17.7 (3.9)	
PANSS general, mean (SD)		35.4 (7.5)	
PANSS total, mean (SD)		69.8 (12.7)	

Abbreviations: IQR, interquartile range; PANSS, Positive and Negative Syndrome Scale.

### Neuroimaging

#### PET Acquisition

Details on PET data acquisition and analysis were previously described (Arumuham et al., 2023). The scan was performed on a Siemens BioGraph 6 HiRez PET-CT scanner (Siemens). All participants underwent a dynamic, continuous, 90-minute PET acquisition after a bolus injection of [^11^C]MK-8278, a radiotracer with high affinity and selectivity for H3R (Van Laere et al., 2014). Scans were performed during the same time period during the day (10:00 am to 1:00 pm). Dynamic PET data were binned in 26 frames according to the following binning: (8 × 15 seconds, 3 × 60 seconds, 5 × 120 seconds, 5 × 300 seconds, 5 × 600 seconds). In parallel to PET imaging, continuous arterial sampling using a blood sampler (Allogg ABSS, Allogg AB, Mariefred, Sweden) was performed for the first 15 minutes followed by 12 discrete samples to measure radiotracer concentrations in blood. A low-dose CT topogram (0.36 mSv) was acquired before PET acquisition for attenuation correction during the PET image reconstruction.

#### PET Analysis

H3R availability was determined as the [^11^C]MK-8278 volume of distribution (V_T_, mL/cm^3^) calculated using the standard 2-tissue compartmental modelling method with a metabolite-corrected arterial plasma input function (Arumuham et al., 2023). Before kinetic modeling, all participants’ PET data underwent a standard image-processing pipeline to correct for subject motion and segment brain tissues. [^11^C]MK-8278 tracer activity was extracted in the ACC and striatum, which were selected as the primary ROIs. These ROIs were defined by the Clinical Imaging Centre neuroanatomy atlas (Tziortzi et al., 2011) using a combination of Statistical Parametric Mapping 12 (http://www.fil.ion.ucl.ac.uk/spm) and Functional Magnetic Resonance Imaging (MRI) of the Brain Software Library (https://fsl.fmrib.ox.ac.uk/fsl/fslwiki/) functions, as implemented in MIAKAT (http://www.imanova.co.uk).

#### MRI and ^1^H-MRS Acquisition

All participants underwent structural MRI to facilitate the anatomical delineation of ROIs. T1-weighted 3-dimensional magnetization-prepared rapid acquisition gradient echo images were acquired on a Siemens Magnetom Verio Syngo MR B17 3T scanner (Siemens) according to the following parameters: repetition time = 2300.0 milliseconds, echo time = 2.98 milliseconds, inversion time = 900 milliseconds, flip angle = 9°, field of view = 256 × 256 mm, 160 sagittal slices of 1-mm thickness, distance factor = 50%, voxel size = 1.0 × 1.0 × 1.0 mm. Magnetization-prepared rapid acquisition gradient echo was acquired to enable ^1^H-MRS voxel prescription and segmentation.

**Figure 1.**
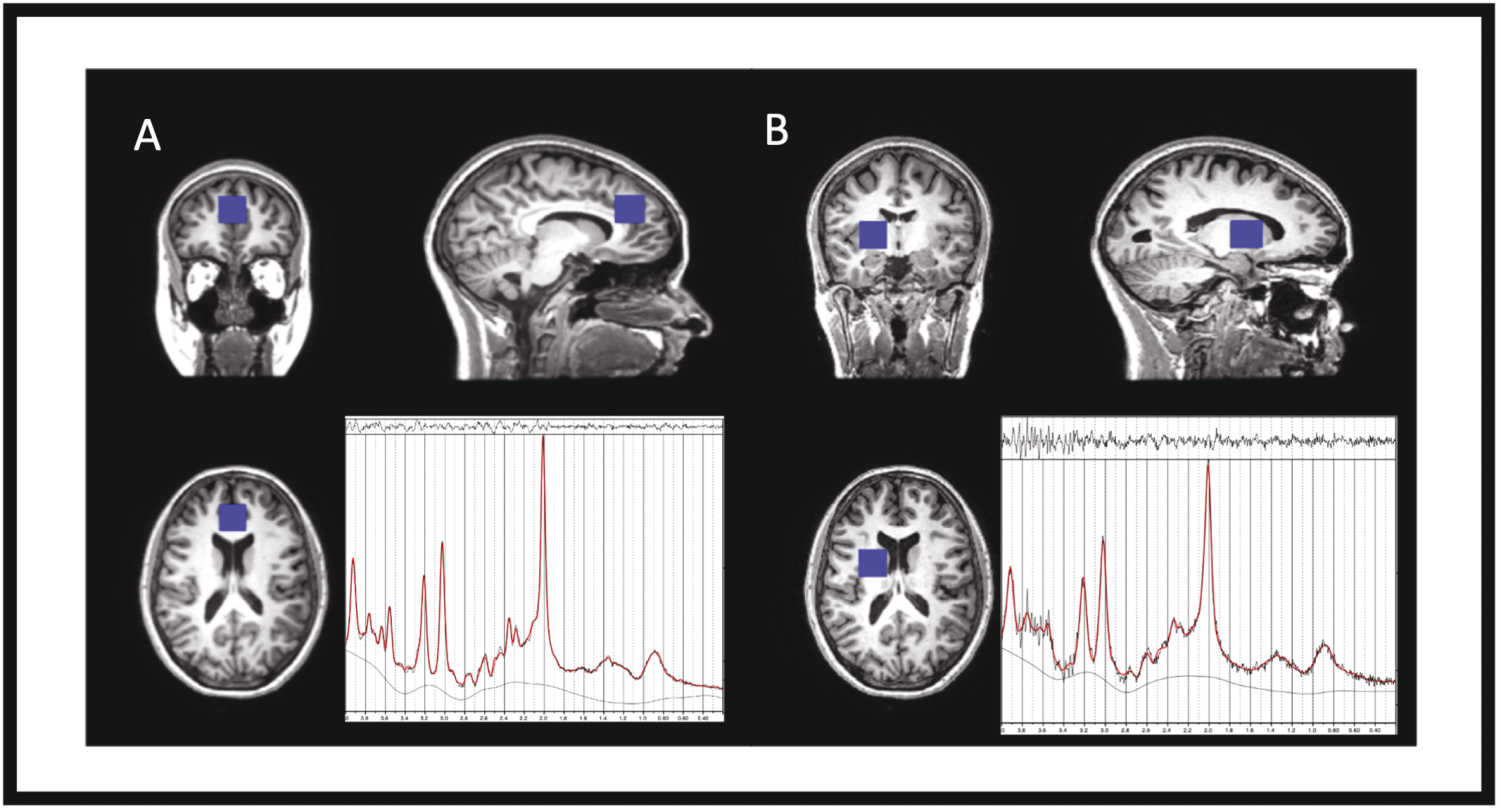
Voxel placement of the (A) ACC and (B) right striatum with corresponding example spectra from ^1^H-MRS. Example spectra are LCModel outputs, with raw data marked in black and fitted data marked in red.

Proton MRS spectra were acquired for both the ACC and the right striatum using a standard PRESS sequence (Point RESolved Spectroscopy, echo time 30 milliseconds, repetition time 3000 milliseconds, number of acquisition points 2048, acquisition bandwidth 2500 Hz, averages 128). Water unsuppressed data were acquired with a separate acquisition with 16 averages. Voxel location for the ACC was determined to include mostly grey matter using sagittal and reformatted coronal images. Voxel dimensions were 2 × 2 × 3 cm^3^ and positioned parallel to and above the corpus callosum, starting from the genu of the corpus callosum and extending 3 cm posteriorly, which was based on a previous study (Bustillo et al., 2014). The striatal voxel (dimensions 2 × 2 × 2 cm^3^) was placed at the lower end of the right dorsal caudate. It was located 3 mm dorsal to the anterior commissure, including the maximum amount of grey matter, with dorsal extension (thickness) of 2 cm (de la Fuente-Sandoval et al., 2013a). We acquired data in the right striatum only because MRS studies have failed to find distinct hemispheric differences in glutamate (Wood et al., 2008; Bustillo et al., 2011) and we had limited scanning time. Moreover, several studies have reported MRS glutamate findings in schizophrenia for the right striatum, so our choice of the right striatum enables comparisons with the existing evidence base (de la Fuente-Sandoval et al., 2011, 2013b; Egerton et al., 2020, 2023; Beck et al., 2022). Voxel placement and example spectra can be seen in Figure 1.

#### 1H-MRS Analysis

LCModel version 6.3-1L (http://s-provencher.com/lcmodel.shtml) was used to estimate the water-scaled Glu (glutamate) (primary outcome measure) and Glx (combined signal of both glutamate and glutamine) concentrations. Eddy current correction was applied, and spectra were visually inspected. Metabolite analyses were restricted to data with a Cramer-Rao lower bound (CRLB) for glutamate ≤20% and signal-to-noise ratio ≥10. CRLB is a reliability indicator, as it is an estimate of the SD of the estimated concentration (Provencher, 2014). Percentage SD values for CRLB were taken from the LCModel output. Statistical Parametric Mapping 12 and Gannet 3.1 were used to identify the amounts of grey matter, white matter, and cerebrospinal fluid in the ^1^H-MRS voxel prescribed in the ACC and striatum. The voxel was corrected for voxel composition based on the formula below (where M = raw metabolite value, GM = grey matter fraction, WM = white matter fraction, CSF = cerebrospinal fluid fraction) (Bloomfield et al., 2021).


$$\begin{array}{l} M_{Corr}=\frac{M*\left(1.207*GM+WM+1.548*CSF\right)}{\left(1-CSF\right)} \end{array}$$

### Statistical Analysis

Statistical Product and Service Solutions (SPSS) version 22 (IBM Corp) was used for all statistical analyses, and the significance level was set to *P* < .05 (2-tailed). Data normality was assessed using the Shapiro-Wilk test. The presence of outliers was assessed using the Tukey method within SPSS (Tukey, 1977). Categorical variables were compared across groups using χ^2^ tests; continuous variables were assessed using independent-samples *t* tests and Mann-Whitney tests for parametric and nonparametric data, respectively.

To test our primary hypothesis that H3R availability is negatively correlated with Glu in the ACC and striatum, we conducted either a Pearson or Spearman correlation for normally distributed and non-normally distributed data, respectively. As the primary outcome, it was corrected for multiple comparisons with a significant threshold value of α < .025 to account for the 2 ROIS (*P* = .05/2: to account for the 2 ROIs). Exploratory analyses were preformed to generate novel hypotheses requiring further testing and thus were not corrected for multiple comparisons. These included analysis to assess whether H3R availability is also negatively correlated with Glx in the ACC and striatum, using either Pearson or Spearman correlation according to the normality of the data. Post hoc comparison of correlation coefficients between patients and controls were performed using a *z*-test on Fisher *r*-to-*z* transformed correlation coefficients (Hinkle et al., 1988).

We performed further exploratory analyses to examine for group differences in H3R availability and glutamate indices.

To examine tracer uptake, a repeated-measures ANOVA was performed, which assessed both the main effect of group status and diagnosis (2: controls, patients) × ROI (2: ACC, striatum) interaction. Independent samples *t* tests were performed post hoc, where appropriate.

Independent *t* tests and Mann-Whitney tests were used, according to normality of data distribution, for comparing group markers of spectral quality (Cramer-Rao Lower Bound, full width at half maximum, signal-to-noise ratio, grey matter [GM] %, white matter [WM] %, cerebrospinal fluid [CSF] %). If significant differences were identified between groups in voxel composition (i.e., GM, WM, and CSF), these parameters were included as covariates in an ANCOVA when determining group differences in the ACC and striatum between cohort groups.

## RESULTS

### Demographics and Experimental Variables

A total of n = 30 participants were recruited for the study, including n = 16 healthy volunteers and n = 14 patients. A total of n = 3 participants (n = 2 healthy volunteers, n = 1 patient) withdrew consent before scanning. Of the remaining participants, n = 3 (n = 2 healthy volunteers, n = 1 patient) withdrew consent following the MRI scan. The final number of participants who received both a PET and ^1^H-MRS scan, and were thus included in the study, was n = 24 participants (n = 12 healthy volunteers, n = 12 patients).

No significant group differences were found for age (U = 67.50, *P* = .80; Mann-Whitney U test) or weight (t_22_ = −1.26, *P* = .22; independent *t* test), and the sex of both groups was matched exactly (see Table 1). On average, patients received 9% less injected dose than controls, but this was not corrected for in further analysis because the injected dose showed no significant correlation with V_T_ values (ACC: Spearman rho = 0.04, *P* = .84; striatum: rho = 0.09, *P* = .67).

### Group Comparison of Imaging Data

#### PET

V_T_ data for both ROIs were normally distributed (ACC: *P* = .46, striatum: *P* = .07; Shapiro-Wilk test of normality), and no outliers were present. We found no statistically significant effect of group on V_T_ in either ROI (ACC: control mean ± SD = 15.7 ± 2.7, patient mean ± SD = 14.2 ± 1.8, striatum: control = 24.6 ± 4.6, patient = 22.3 ± 5.1, main effect of group F_1,21 _= 1.98, *P* = .18; repeated measures ANOVA), and there was no group-by-ROI interaction (F_1,21_ = 0.30, *P* = .59).

#### 1H-MRS

Metabolite concentrations, spectral quality, and voxel segmentation for ACC and striatum voxel acquisition are shown in Tables 2 and 3 respectively.

**Table 2.** Voxel segmentation and spectral quality in the ACC

ACC	x			
Parameter	Controls/ mean (SD)	Patients/ mean (SD)	Effect size	*P*
Cramer-Rao lower bound—Glu, %	9.86 (0.88)	9.97 (1.13)	^*^*^ *Cohen’s d* = 0.11	.79
Cramer-Rao lower bound—Glx, %	12.96 (1.31)	12.67 (1.69)	^*^*^ *Cohen’s d* = 0.03	.64
Full width at half maximum, ppm	0.04 (0.01)	0.05 (0.02)	^*#*^ *r =* 0.14	.51
Gray matter, %	63.12 (5.63)	62.31 (4.71)	^*^*^ *Cohen’s d* = 0.16	.71
White matter, %	17.65 (4.77)	13.43 (3.48)	^*^*^ *Cohen’s d* = 1.01	.02*
Cerebrospinal fluid, %	19.23 (4.59)	24.26 (6.14)	^*^*^ *Cohen’s d* = 0.87	.04*

Abbreviations: ^^^, independent samples *t* test; ^#^, Mann-Whitney U test; *, *P* < .05; ppm, parts per million. Cramer-Rao lower bound is the lower bound estimate of the standard deviation of the estimated metabolite concentration. Full width at half maximum is the spectral width at the half amplitude of the signal and is a marker of spectral quality.

**Table 3.** Voxel segmentation and spectral quality in the striatum

Striatum	x			
Parameter	Controls/ mean (SD)	Patients/ mean (SD)	Effect size	P
Cramer-Rao lower Bound—Glu, %	6.57 (0.70)	5.99 (1.33)	^*#*^ *r =* 0.26	.27
Cramer-Rao lower bound—Glx, %	9.80 (2.05)	8.88 (1.86)	^*^*^Cohen’s d = 0.74	.26
Full Width at half maximum, ppm	0.08 (0.02)	0.10 (0.02)	^*#*^ *r =* 0.42	.05
Gray matter, %	53.33 (5.54)	57.54 (2.94)	^*^*^Cohen’s d = 0.95	.03*
White matter, %	45.49 (5.35)	41.29 (2.10)	^*^*^Cohen’s d = 1.04	.02*
Cerebrospinal fluid, %	1.18 (1.18)	1.17 (1.49)	^*#*^ *r =* 0.09	.67

Abbreviations: ^, independent samples *t* test; #, Mann-Whitney U test; *, *P* < .05; ppm, parts per million. Cramer-Rao lower bound is the lower bound estimate of the standard deviation of the estimated metabolite concentration. Full width at half maximum is the spectral width at the half amplitude of the signal and is a marker of spectral quality.

In the ACC we did not find any outliers for Glu but did identify one in the patient group for Glx (Gl = 9.01, mean [SD] = 12.96 [1.31], IQR = 1.90). For measures of Glu in the striatum, we found 1 outlier in the control group (Glu = 5.12, mean [SD] = 6.57 [0.70], IQR = 0.84) and 1 in the patient group (Glu = 2.93, mean [SD] = 5.99 [1.33], IQR = 1.71). Finally, we found an outlier for Glx in the striatum in the patient group (Glx = 13.08, mean [SD] = 8.88 [1.86], IQR = 2.19). These outliers were excluded from further analysis. Upon removal of outliers, all MRS metabolite values were normally distributed.

In the ACC, voxel segmentation analysis identified a significant difference in proportional voxel composition between patients and controls in WM (t = 2.47, *P* = .02) and CSF (t = −2.14, *P* = .04). When including WM and CSF as a covariate in a between-group ANCOVA, we did not find a significant difference between patients and controls for Glu (F_1,20_ = 2.02, *P* = .17; ANCOVA). However, we found a significant effect of group on ACC Glx when including WM and CSF as covariates (F_1,19 _= 4.75, *P* = .04).

In the striatum, there were significant differences in proportional voxel composition for GM (t = −2.33, *P* = .03) and WM (t = 2.54, *P* = .02). When these parameters were included as covariates in a between-group ANCOVA, we found a significant difference between patients and controls for measures of Glx (F_1,20 _= 6.00, *P* = .02), but not for Glu (F_1,20_ = 3.03, *P* = .10).

#### Correlation Between [11C]MK-8278 VT and 1H-MRS Measures of Glutamate

**Figure 2.**
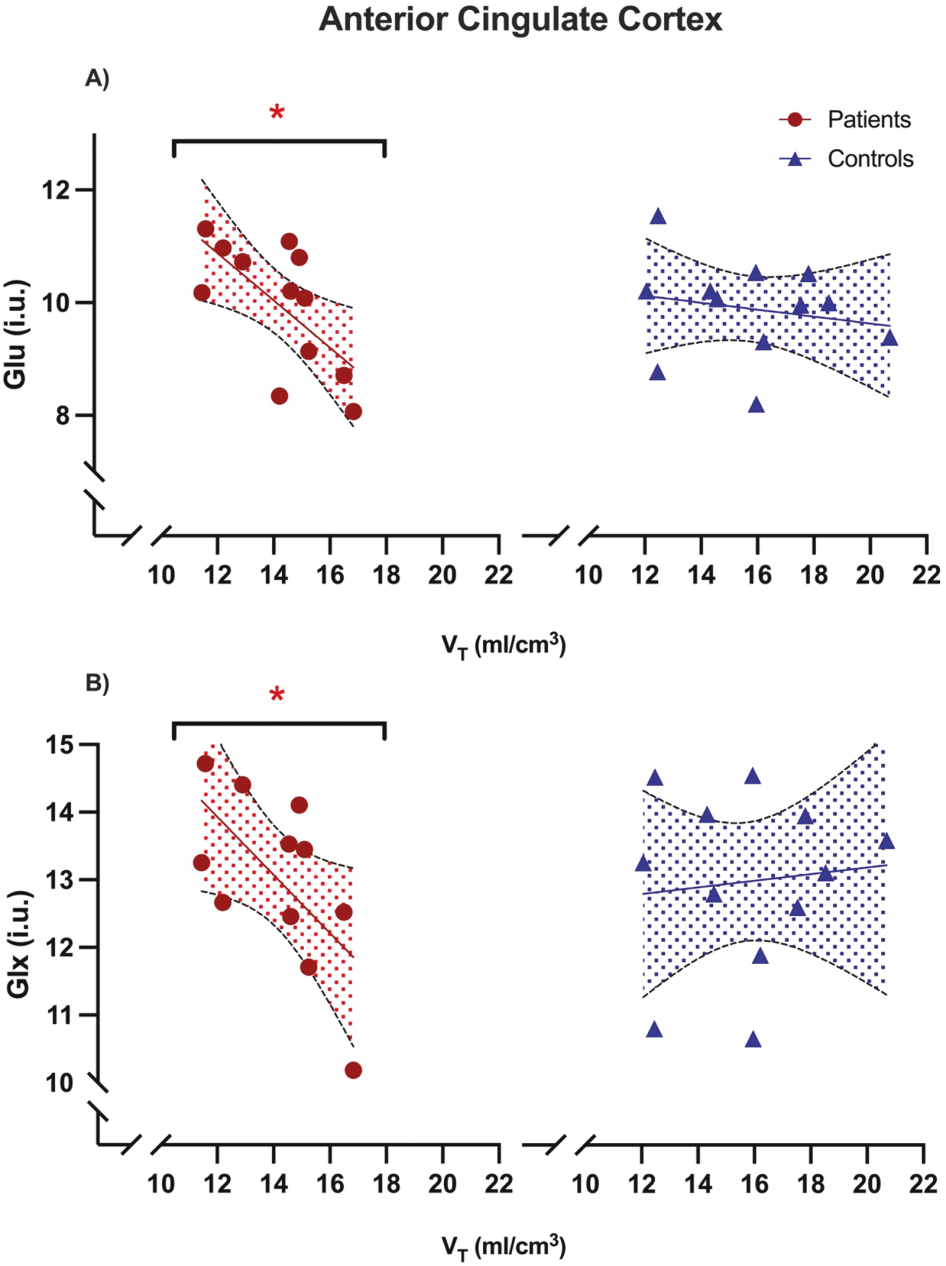
Relationship between H3R availability and glutamate levels. H3R availability measured as [^11^C]MK-8278 tracer uptake and glutamate levels measures as Glu and Glx collected through ^1^H-MRS in the ACC. (A) Portrays measures of Glu in both patients and controls, while (B) portrays measures of Glx in both patients and controls. The shaded areas indicate 95% CIs. *: statistically significant.

Testing our primary hypothesis in the ACC, we found a significant negative relationship between VT and MRS measures of Glu (r = −0.66, α = .019; Pearson correlation) in patients but not in healthy controls (r = −0.19, α = .560) (Figure 2). Comparison of correlation coefficients did not find a significant difference between groups (rdiff = −0.47, z = −1.27, 95% CI = −0.91 to 0.31, *P* = .200, Fisher r-to-z, 2-tailed). Similarly, for our exploratory analyses in the ACC, we found a significant negative relationship between VT and Glx in patients (r = −0.62, *P* = .042) but not in controls (r = 0.10, *P* = .75), without a significant difference of correlation coefficients between groups (rdiff = −0.72, z = −1.70, 95% CI = −0.94 to 0.13, *P* = .090).

Conversely, in the striatum, we found no significant relationship between V_T_ and Glu (patients: r = 0.47, *P* = .174, controls: r = 0.23, *P* = .501), or V_T_ and Glx (patients: r = 0.33, *P* = .357; controls r = −0.56, *P* = .061) in either group.

## DISCUSSION

Our primary finding was a significant inverse relationship between ACC [^11^C]MK-8278 tracer uptake and MRS measures of Glu in patients, which is absent in healthy controls. Exploratory analyses found a similar negative correlation between ACC H3R availability and Glx in patients alone. Comparison of correlations did not identify a significant difference between groups for these associations. We also report a significant difference between patients with schizophrenia and controls for ACC and striatal Glx, with patients found to have lower mean Glx concentrations.

To our knowledge, this is the first in vivo study to characterize the relationship between H3R availability and glutamate levels in humans, and our findings of an inverse relationship extends existing preclinical evidence implicating the role of H3R in glutamate release (Molina-Hernández et al., 2001; Takei et al., 2017). The ACC, which is associated with several cognitive processes (Richard et al., 2004; Vincent et al., 2008), has been found to have alterations of the glutamate system in schizophrenia depending on illness progression (Jauhar et al., 2018). Meta-analytic studies have identified lower glutamate levels in voxels, including the ACC of patients with schizophrenia compared with healthy volunteers (Marsman et al., 2013; Merritt et al., 2023). Moreover, studies have found that lower glutamate metabolite levels in the ACC of patients with schizophrenia are correlated to poorer cognitive task performance (Ohrmann et al., 2008; Bojesen et al., 2021; Coughlin et al., 2021; Griffiths et al., 2022). The findings that we report, together with existing evidence of lower ACC glutamate levels being associated with impaired cognition, suggest that H3R antagonism in patients with schizophrenia may improve cognitive impairment, which is a feature of the disorder, by increasing glutamate transmission. Consistent with this, betahistine (H1R agonist/H3R antagonist) has been found to improve cognitive symptoms (Wang et al., 2021). However, H3R-specific compounds have shown mixed results in treating cognitive impairment associated with schizophrenia in clinical trials (Vohora and Bhowmik, 2012; Egan et al., 2013; Haig et al., 2014; Jarskog et al., 2015).

It is interesting to note that the relationship between ACC tracer uptake and glutamate levels was only found in patients and not in healthy volunteers. In part, this may be due to the functional properties of H3R. Notably, H3R has high constitutive activity, with higher expression of the receptor associated with reduced synthesis and release of neurotransmitters (Schlicker et al., 1994; Morisset et al., 2000; Moreno-Delgado et al., 2006; Arrang et al., 2007; Zheng et al., 2023). There are 20 H3R isoforms that have been identified and found to have differential expression and functionality across the CNS (Cogé et al., 2001; García-Gálvez and Arias-Montaño, 2016). However, the expression of H3R isoforms has not yet been studied in psychotic disorders. Moreover, the roles of different functional isoforms in glutamate transmission are unknown. Thus, functional differences of H3Rs in patients with schizophrenia compared with healthy controls could be a hypothetical cause of only identifying a significant relationship between H3R availability and glutamate levels in patients. However, to elucidate these ideas, further studies using drug challenges of H3R antagonists would be beneficial to understand potential variation in the functional properties of H3Rs and its effect on glutamate in both disease and healthy states.

Our findings also identified the lack of relationship between H3R and glutamate levels in the striatum. Mapping of H3Rs in preclinical studies has found that the striatum contains the highest levels of the receptor in the CNS (Pollard et al., 1993; Pillot et al., 2002). As well as a presynaptic receptor, H3R has been localized postsynaptically, where it interacts with other receptors (such as dopaminergic receptors) to modulate downstream signaling (Ferrada et al., 2008, 2009; Moreno et al., 2011; Xu and Pittenger, 2023). Studies have identified the majority of H3Rs localized in the striatum are found postsynaptically, and thus a much lower proportion act as heteroreceptors on afferent pathways, such as corticostriatal neuron terminals (Ryu et al., 1994; Pillot et al., 2002). The more abundant postsynaptic H3Rs in the striatum are largely found on medium spiny neurons, where they interact with D1 dopamine receptors of the direct pathway and D2 dopamine receptors of the indirect pathway (Ferrada et al., 2008, 2009; Ellenbroek and Ghiabi, 2014; Bolam and Ellender, 2016). Conversely, the lower proportion of presynaptic H3Rs found on the corticostriatal pathway cause reductions in glutamate release, thus driving a suppressing effect on pathway signaling (Ellender et al., 2011; González-Sepúlveda et al., 2013). These preclinical findings, taken together with our findings of a lack of significant relationship between striatal glutamate and H3R levels, could indicate that humans share a similar mapping of striatal H3R. Namely, that the majority of H3R localized in this region is likely to be postsynaptic and thus unlikely to influence clinically significant glutamate release in the striatum.

Studies have found that patients with schizophrenia have higher levels of striatal glutamate levels compared with healthy volunteers (Merritt et al., 2016; Nakahara et al., 2022). Speculatively, this suggests that H3R antagonists may have interesting properties in the treatment of schizophrenia. One hypothesis is that the regional specificity of H3R antagonists on glutamate release might allow H3R antagonists to increase cortical glutamate transmission while sparing striatal levels. In turn, this may potentially improve cognition while not exacerbating positive symptoms of schizophrenia, where the latter may be influenced by elevated striatal glutamate.

We found a group difference for both ACC and striatal Glx, which is similar to findings from previous studies, although this group difference in ROIs was not found for Glu (Marsman et al., 2013; Merritt et al., 2016, 2023; Nakahara et al., 2022). Glutamine is synthesized through the astrocytes metabolizing synaptic glutamate and thus can be considered a marker of glutamate neurotransmission (McCutcheon et al., 2020a). Thus, our findings may suggest reduced transmission and subsequent metabolism of glutamate to glutamine. Because our study was not powered to detect a group difference in ^1^H-MRS estimates of glutamate metabolites, it is also possible that this is due to a Type II error. However, our results add to existing literature that has shown mixed findings regarding glutamate levels in patients with schizophrenia (Merritt et al., 2023). There is meta-analytic evidence that indicates antipsychotic exposure may have a stronger impact on variations in glutamate rather than age or disease progression, namely driving a reduction in glutamate levels (Merritt et al., 2021; Nakahara et al., 2022). Because our patient group was made up of medicated and unmedicated participants (n = 7 and n = 5, respectively), we are unable to exclude a potential confounding effect of antipsychotic medications on glutamate concentrations. Although the sample size is small, we found no significant relationship between chlorpromazine-equivalent doses of antipsychotic treatment and ACC (rho = 0.46, *P* = .13) or striatal glutamate levels (rho = −0.21, *P* = .53), suggesting a limited effect of antipsychotic exposure in our results.

### Strengths and Limitations

A notable strength in this study is that the radioligand [^11^C]MK-8278 has a high affinity for H3R. When evaluated in more than 170 receptor binding or enzyme assays, the compound was found to be selective for H3R (Ki = 0.54 nM), with only weak off-target binding found at 5-HT_2_ (51% inhibition at 10 μM) and 5-HT_2A_ (62% inhibition at 10 μM). The tracer has shown reliability in providing accurate V_T_ estimates by having a test-retest variation of approximately 5% in most regions (Van Laere et al., 2014). However, there may be a limitation in interpreting tracer uptake in the striatum, because this study also found increased variation of uptake in the caudate where repeatability was 20%. A further strength is that our patient sample involved people in the earlier stages of their illness, many of whom were antipsychotic free/naïve. Therefore, illness duration and chronic antipsychotic treatment are unlikely to have influenced the observed association. Furthermore, given the diurnal variation of histamine release in the CNS (Brown et al., 2001), we limited potential temporal confounding by ensuring scans for all participants were performed during the same period of time during the day.

As mentioned above, limitations of the study include a modest sample size and the concurrent use of antipsychotic medication in our patient group. Further limitations in the use of ^1^H-MRS imaging also need to be considered when interpreting results. Firstly, the modality is unable to distinguish between intra- and extracellular compartments (Poels et al., 2014). Glutamate also plays roles in protein synthesis, nitrogen metabolism, and GABA synthesis (Bak et al., 2006). As a result, differences observed between patients and controls cannot be confidently attributed to variations in synaptic transmission of glutamate levels alone but could also stem from other glutamatergic functions that H3R is not known to have a role in. Preclinical data has shown H3R influence synaptic glutamate release (Molina-Hernández et al., 2001; Takei et al., 2017), although there is no current evidence implicating its role in glutamate synthesis. In view of this, discrimination of intracellular and extracellular compartments is necessary to clarify the influence of H3R on glutamate concentrations. Higher field strengths would be of benefit for use in future studies, because it can differentiate between glutamate and its metabolite glutamine, whereas lower strengths can only accurately quantify the concentration of both (Snyder and Wilman, 2010). Moreover, characterizing H3R with functional MRS measures of glutamate would provide further information on task evoked changes in glutamate levels (Jelen et al., 2018). It is important to note that the design of the study limits the interpretation of findings to correlation. To address this, further studies are necessary to employ specific H3R modulating drug challenges to determine the direction of the relationship.

## CONCLUSIONS

Our findings provide evidence for an inverse relationship between ACC H3R and glutamate levels in patients with schizophrenia. We found no evidence of a significant relationship between H3Rs and glutamate in the striatum. Our findings implicate H3R in glutamate regulation in a cortical region strongly associated with cognitive processes such as working memory, executive function, and reward-based learning. Future studies focusing on drug challenges using H3R modulating compounds and imaging at higher field strength would provide further details on the relationship highlighted in our work. Nevertheless, these results build on a body of evidence demonstrating the involvement of H3Rs in glutamate neurotransmission and potentially highlight a novel target to treat cognitive symptoms of schizophrenia.

## Supplementary Material

pyae011_suppl_Supplementary_Material

## Data Availability

The data that support the findings of this study are available from the corresponding authors, AA and ODH, upon reasonable request.

## Interest Statement

AA, MMN, MV, KB, ECO, and DJL have no conflicting interests to declare. SJ has received honoraria for educational talks given for Boehringer Ingelheim, Lundbeck, Janssen and Sunovion, has advised on antipsychotics to LB Pharmaceuticals, and has sat on a Wellcome Funding Panel on backwards translation in mental health. EAR is a full-time employee of Invicro, an imaging centre conducting contract studies for pharmaceutical and biotech partners. As part of his role, EAR has consulted for numerous industry and academic entities. ODH is a part-time employee and stockholder of Lundbeck A/s. He has received investigator-initiated research funding from and/or participated in advisory/ speaker meetings organised by Angellini, Autifony, Biogen, Boehringer-Ingelheim, Eli Lilly, Heptares, Global Medical Education, Invicro, Jansenn, Lundbeck, Neurocrine, Otsuka, Sunovion, Recordati, Roche and Viatris/ Mylan. ODH has a patent for the use of dopaminergic imaging.
