# Supplementary material for: Histamine-3 Receptor Availability and Glutamate Levels in the Brain: A PET-1H-MRS Study of Patients With Schizophrenia and Healthy Controls
Source: Int J Neuropsychopharmacol. 2024 Feb 19;27(3):pyae011. doi: 10.1093/ijnp/pyae011 (PMC10946236; doi:10.1093/ijnp/pyae011)
Supplement: pyae011_suppl_Supplementary_Material [file pyae011_suppl_supplementary_material.docx]

**Supplementary Information**

**s-Methods 1: Inclusion and exclusion criteria**

Inclusion criteria for all participants: 1) between 18­65 years of age; 2) capacity to consent to participation in the study; 3) modified Allen’s test showing adequate collateral circulation to the hand (to ensure safety of radial arterial catheterization); 4) no history of clotting or renal abnormality and no abnormal blood results on screening blood test; 5) English speaking sufficient to understand task instructions and information sheet; 6) in females, a negative urine pregnancy test at screening visit, and before PET and MRI scan. 7) for patients, a Diagnostic and Statistical Manual of Mental Disorders (DSM-IV) diagnosis of a schizophrenia according to the *Structured Clinical Interview of DSM-IV-TR Axis I Disorders-Patient Edition*.

Exclusion criteria for all participants: 1) ages <18 or >65; 2) a history of a head injury resulting in loss of consciousness; 3) personal history of serious medical illness; 4) contraindication to MRI scanning (e.g., metallic implants, claustrophobia, inability to lie comfortably for 90 minutes); 5) radiation exposure that would take total exposure (including participation in this study) to >10mSv in 12 months; 6) current or lifetime history of substance use or dependence as determined by the Structured Clinical Interview for DSM-IV-TR (SCID-I/P); 7) screened positive for any of the following substances (except cannabis) on a multi-panel urine drug screen detecting the following substance; amphetamine (300ng/ml cut off), cocaine (150 ng/ml cut off), ketamine (1000 ng/ml cut off), cannabis (50ng/ml cut off), methamphetamine (300 ng/ml cut off), opiates (2000 ng/ml cut off) (SureScreen Diagnostics, Derby); 8) current or recent use (no use within 3 months) of H3R modulating drugs; 9) donation of blood or blood products in excess of 500ml within a 60 day period prior to the present study; 10) for healthy volunteers, a lifetime history of an Axis-I psychiatric disorder (DSM-IV) or confirmed diagnosis of Axis-I disorder in 1^st^ degree relatives.

**s-Figure 1: Striatal PET - ^1^H-MRS data correlations**

**
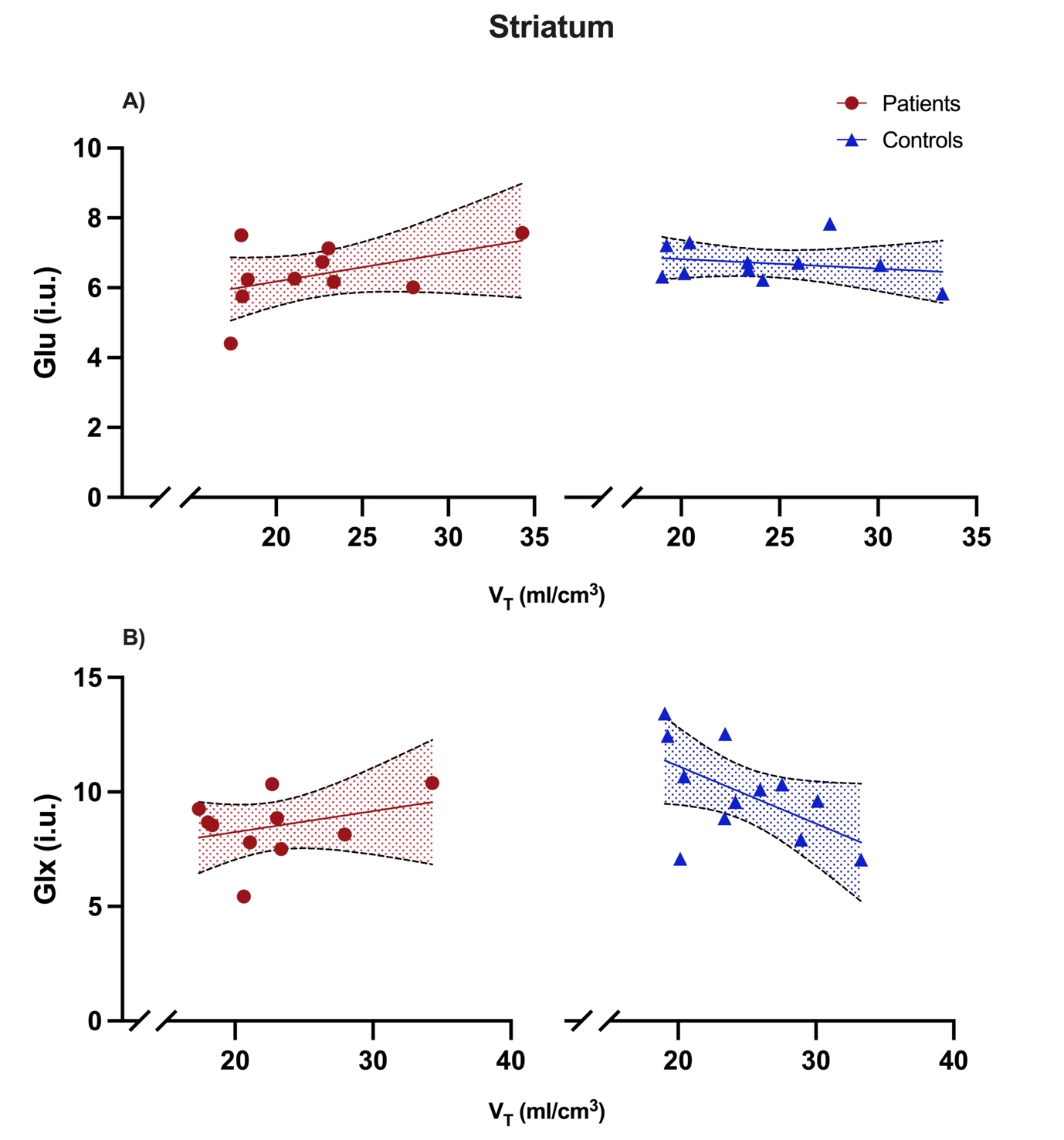
**

Graphical representation of the relationship between [^11^C]MK-8278 tracer uptake and measures of glutamate collected using ^1^H-MRS in the striatum. A) portrays measures of Glu in both patients and controls, while B) portrays measures of Glx in both patients and controls. The shaded areas indicate 95% confidence intervals.
